# Efficient synthesis of 4-substituted-*ortho*-phthalaldehyde analogues: toward the emergence of new building blocks

**DOI:** 10.3762/bjoc.15.67

**Published:** 2019-03-19

**Authors:** Clémence Moitessier, Ahmad Rifai, Pierre-Edouard Danjou, Isabelle Mallard, Francine Cazier-Dennin

**Affiliations:** 1 Unité de Chimie Environnementale et Interactions sur le Vivant (UCEIV) - Université du Littoral Côte d'Opale, 145 Avenue Maurice Schumann, MREI 1, 59140 Dunkerque, France; 2 Lebanese Atomic Energy Commission – National Council for Scientific Research – B. P. 11- 8281, Riad El Solh 1107, 2260 Beirut, Lebanon

**Keywords:** demethylation, hydroxy group protection, 4-hydroxy-*ortho*-phthalaldehyde, *ortho*-phthalaldehyde, oxidation

## Abstract

4-Methoxy-*ortho*-phthalaldehyde and 4-hydroxy-*ortho*-phthalaldehyde are potentially useful molecules for fluorimetric analysis of a variety of amines and for the elaboration of complex molecular architectures. Nevertheless, literature generally describes their synthesis in very low yield (below 5%), mainly due to the inefficiency of the last oxidation step. In this paper, we report a reliable synthesis of 4-substituted-*ortho*-phthalaldehyde analogues in 51% overall yield owing to the addition of a protecting step of the unstable key intermediate 4,5-dihydroisobenzofuran-5-ol. Oxidation and deprotection steps were also studied in order to provide an effective availability of these two dialdehyde compounds that may increase their future applications.

## Introduction

Benzene-1,2-dicarboxaldehyde, also called *ortho*-phthalaldehyde (OPA) was employed in different domains such as in analytical and organic chemistry. In fact, it is one of the most commonly used dialdehydes in fluorometric methods with regard to the analysis of amino acids and biogenic amines [1–3]. It is also used as a derivatization reagent in order to quantify hydrazine by gas chromatography coupled with a mass spectrometer detector [4]. Recently, it was adopted for the analysis of γ-aminobutyric acid by high-performance liquid chromatography with molecular fluorescence detection [5]. In addition to the above manipulations, OPA and its derivatives are also valuable reagents [6] in organic chemistry, used to generate phthalimidine [7], imines [8], isoindole [9], 3-hydroxyindanone [10], thioether macrocycles [11], macrocyclic Schiff bases [12] or even poly(phthalaldehyde) polymers [13]; taking into account that few studies were carried out with modified OPA on position 3 or 4. This could probably be due to the fact that the synthesis of 4-substituted OPA was, to the best of our knowledge, rarely recorded in the literature with an efficient synthetic pathway; conjointly to the 3-substituted OPA that is even less described.

In 1966, Khan et al. presented the synthesis of 4-hydroxy and 4-methoxy-*ortho*-phthalaldehyde (4-HO-OPA and 4-MeO-OPA) by bromination and oxidation of 4-hydroxy and 4-methoxy-*ortho*-xylene in only 5% yield [14]. 4-HO-OPA was also described in 1997 by Taylor et al. as a crude product (via a Diels–Alder reaction of commercially available Danishefsky diene with 4,4-diethoxybut-2-ynal) for the synthesis of an antitumor analogue [15]. This aforementioned strategy was applied as well for the preparation of hydantoin derivatives to treat anti-inflammatory disorders [16]. More recently, in a study of Cao et al. [17] 4-HO-OPA was synthesized, once again in a low yield (4%) in order to produce the cucurbit[*n*]uril core.

As shown by literature, the poor efficiency of 4-substituted OPA synthesis represents a major limiting factor to their potential applications. Herein, we set forth a new protection-deprotection strategy which leads to 4-MeO-OPA and 4-HO-OPA as reliable structures for organic and analytical chemistries. A variety of protecting groups of key 4,5-dihydroisobenzofuran-5-ol intermediate has been tested and oxidation parameters of the dedicated structures were optimized. Finally, a rapid deprotection step was initiated to afford 4-MeO-OPA and 4-HO-OPA with 51% overall yield.

## Results and Discussion

**Scheme 1:**
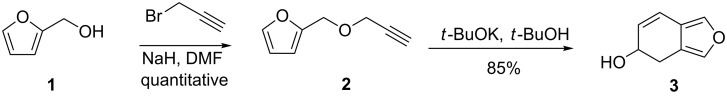
Synthesis of 4,5-dihydroisobenzofuran-5-ol (**3**).

Firstly, 2-((prop-2-ynyloxy)methyl)furan (**2**) was synthesized through a reaction of furfuryl alcohol with propargyl bromide, as is referred in the study of Cao et al. (Scheme 1) [17]*.* As a matter of fact, a modification of the purification step was done in order to quantitatively enhance the yield. An alternative solvent-free microwave-assisted organic synthesis (MAOS) protocol for **2** was also tested in a basic medium with tetrabutylammonium bromide [18]. Nevertheless, this convenient time-saving procedure (15 minutes) shows a lower 74% yield due to the presence of a byproduct (propargylic alcohol) as evidenced by NMR spectroscopy. An intramolecular Diels–Alder cyclisation in a basic medium of **2** then occurred to generate 4,5-dihydroisobenzofuran-5-ol (**3**) [19].

At this step, Cao et al. [17] have chosen the direct oxidation of 4,5-dihydroisobenzofuran-5-ol (**3**) to obtain 4-HO-OPA by using 2,3-dichloro-5,6-dicyano-1,4-benzoquinone (DDQ) as an oxidant. However, the yield of this reaction was very low (only 13%) mainly due to major production of OPA as is also described in a study of Wenkert et al. [20]. In order to circumvent the dehydration of **3** which generate OPA under oxidative conditions, a protection strategy of the hydroxy group was undertaken.

**Scheme 2:**
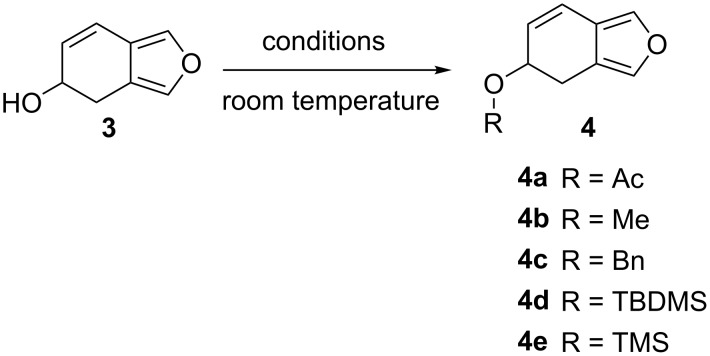
Protection strategy of 4,5-dihydroisobenzofuran-5-ol (**3**).

The only protecting group described in the literature for 4,5-dihydroisobenzofuran-5-ol (**3**) is acetyl [19]. Thus, beside this former, several protecting groups such as: methyl (Me), benzyl (Bn), *tert*-butyldimethylsilyl (TBDMS) and trimethylsilyl (TMS) were tested (Scheme 2). As summarized in Table 1, major disparity in the yield was observed, ranging from 95% for methyl and acetyl to only 10% for benzyl. The poor yield for standard protection conditions could be explained by the sensitivity of compound **3** to reflux conditions. Based on these results, acetyl and methyl have been selected for the protection. It should be mentioned that 5-acetoxy- and 5-methoxy-4,5-dihydroisobenzofuran appear to be shelf stable at room temperature and can be stored for at least one month in contrast to the 5-hydroxy derivative which is liable to degradation.

**Table 1:** Protection of 4,5-dihydroisobenzofuran-5-ol (**3**).

R	reactant	solvent	yield (%)

Ac	Ac_2_O	pyridine	95
Me	MeI/NaH	anhydrous dioxane	95
Bn	BnCl/NaH	anhydrous dioxane	10
TBDMS	TBDMSCl/imidazole	anhydrous THF	30
TMS	TMSCl/imidazole	anhydrous THF	15

**Scheme 3:**
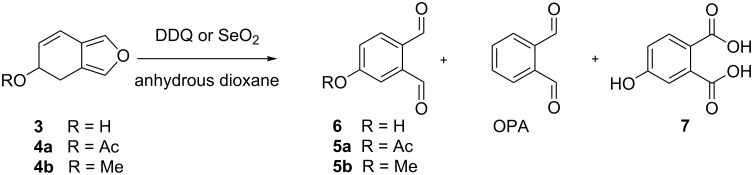
Oxidation of 5-substituted-4,5-dihydroisobenzofuran-5-ol in presence of SeO_2_ or DDQ.

Following the protection of the hydroxy group, the oxidation step was initiated in order to evaluate the stability of the protected RO-OPA and to certify the absence of OPA contamination [20]. Two major oxidants, SeO_2_ and DDQ were chosen for 5-methoxy and 5-acetoxy-4,5-dihydroisobenzofuran oxidation. As illustrated in Scheme 3, the reaction occurred with both reactants but ended up with variable amounts of OPA, the desired 4-substitued *ortho*-phthalaldehyde and the phthalic acid analogues **7**.

Quantitative analyses were carried on the ^1^H NMR on the crude mixture and the percentage of each compound was determined by integration of aldehydic or acidic protons.

As indicated in Table 2, the results clearly show that the oxidation of **3** with SeO_2_ (Table 2, entry 1), yields OPA as a main product which is consistent with the study of Wenkert et al. [20]. Similar results were observed for the oxidation of 5-acetoxy-4,5-dihydroisobenzofuran (**4a**) that gave 22% of 4-acetoxy-*ortho*-phthalaldehyde (**5a**) and 78% of OPA (Table 2, entry 2). As for 5-methoxy-4,5-dihydroisobenzofuran (**4b**), it was totally converted to 4-hydroxyphthalic acid (**7**, Table 2, entry 3). In regard to these results, oxidation with SeO_2_ leads to the unwanted products OPA and **7**, so oxidation with DDQ was initiated to avoid side products.

**Table 2:** Ratio quantity of OPA formed during the oxidation of 5-substituted-4,5-dihydroisobenzofuran-5-ol on SeO_2_ or DDQ.

entry	starting product	oxydant	ratio of quantity (%)		

			RO-OPA	OPA	compound **7**

1	**3** R = H	SeO_2_	0	80	20
2	**4a** R = Ac	SeO_2_	22	78	–
3	**4b** R = Me	SeO_2_	0	0	100
4	**3** R = H	DDQ	50	50	–
5	**4a** R = Ac	DDQ	0	100	–
6	**4b** R = Me	DDQ	97	3	–

DDQ oxidation of 4,5-dihydroisobenzofuran-5-ol (**3**) led to identical results to the literature and formed a 1:1 mixture of 4-HO-OPA (**6**) and OPA (Table 2, entry 4). In the case of 5-acetoxy-4,5-dihydroisobenzofuran (**4a**), the oxidation prompts the total formation of OPA (Table 2, entry 5). To our delight, the remarkable stability of the methoxy group under the same synthesis conditions introduces the key intermediate 4-methoxyphthalaldehyde (**5b**) with an excellent conversion rate and minimal OPA formation (Table 2, entry 6). Compound **5b** was then isolated by column chromatography in 63% yield.

After completing the oxidation step with convincing results, the final step of the methoxy group removal was undertaken. Several studies showed that OPA reacts in the presence of water to form a cyclic 1,3-phthalandiol [21] or a dimer [22]. Taking this into account to access pure 4-HO-OPA, it was decided to go for anhydrous deprotection reaction, and in doing so, keeping the dialdehyde part untouched.

Accordingly, hydrogen bromide was employed as a deprotection agent, as previously used by Borisenko et al. [23], to finally achieve the desired product **6** but in a very poor yield (5%). A second deprotection agent was tested, trimethylsilyl iodide, as it was recently published by Danjou et al. [24] as a removal agent of methoxy groups on calixarenes architectures. Although we succeeded with the removal of methoxy groups, both aldehydes were reduced.

Furthermore, the lithium chloride/*N*,*N*-dimethylformamide system was tested according to the study of Fang et al. [25] revealing that the methoxy group in the *meta-*position of an electron-withdrawing substituents can be removed under microwave irradiation. However, when testing the system on **5b**, no reaction occurred. Another experiment with lithium chloride/*N*,*N*-dimethylformamide in the presence of a catalytic amount of *p*-toluenesulfonic acid ended up with only the formation of OPA.

**Scheme 4:**
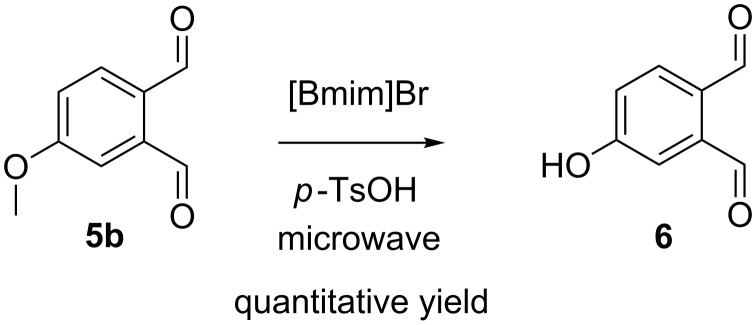
Synthesis of 4-hydroxy-*ortho*-phthalaldehyde (**6**) through MAOS demethylation of 4-methoxy-*ortho*-phthalaldehyde (**5b**).

Regeneration of phenols from methyl ethers have also been tested by Boovanahalli et al. [26] by using an ionic liquid such as 1-butyl-3-methylimidazolium bromide ([Bmim]Br) in the presence of different Brønsted acids. While investigating these conditions in combination with microwave irradiation (Scheme 4), the desired compound 4-HO-OPA (**6**) was successfully obtained in only 30 minutes, with a good yield (75%) when reacting with methanesulfonic acid (MsOH) as catalyst and a quantitative yield upon its reaction with *p*-toluenesulfonic acid (*p*-TsOH).

## Conclusion

This study presents the highest reported yield for the synthesis of 4-methoxy-*ortho*-phthalaldehyde (**5b**) and 4-hydroxy-*ortho*-phthalaldehyde (**6**). For the latter, our study highlights the necessity to protect the key intermediate 4,5-dihydroisobenzofuran-5-ol (**3**) before reaching the oxidation step. The methoxy protecting group and the DDQ oxidation reagent were found to be the most efficient. Moreover, deprotection of 4-methoxy-*ortho*-phthalaldehyde (**5b**) was successfully achieved using [Bmim]Br and *p*-TsOH on MAOS conditions. All the above steps constitute a straightforward approach for the synthesis of 4-methoxy and 4-hydroxy-*ortho*-phthalaldehyde. The effective availability of both these structures may now increase their application as building blocks for future organic entities. In particular, the presence of the versatile polar anchoring group can lead to surface modification inducing a growing interest in these *ortho*-phthalaldehyde analogues for further application.

## Experimental

### Materials and methods

All the chemicals were purchased from Acros Organic or Sigma-Aldrich and were used as received without further purification. Microwave-assisted organic synthesis was carried out using a 400 W Biotage initiator oven. Samples were placed in a sealed vial and irradiated with a temperature control. Automated flash chromatography was performed on a Puriflash 430 (Interchim) incorporating a quaternary pump system, diode array and evaporation light scattering detectors using SiO_2_ HC prepacked cartridge. NMR spectra were recorded at 300 K with a Bruker Ascend 400 spectrometer operating at 400 MHz and 101 MHz for ^1^H and ^13^C, respectively. Traces of residual solvent were used as internal standard.

### Synthetic procedures

**2-((Prop-2-ynyloxy)methyl)furan (2). Adapted from reference** [17]**:** Furfuryl alcohol (9.0 mL, 103.8 mmol) was added dropwise to a solution of NaH (5.0 g, 207.5 mmol) in DMF (100 mL) at 0 °C. After 90 minutes, propargyl bromide (12.7 mL, 114.2 mmol) was added dropwise and the resulting mixture was stirred overnight at room temperature. Water was then added to the solution. The aqueous phase was extracted several times with Et_2_O until a colorless organic phase was obtained. The combined organic phases were washed with brine, dried over MgSO_4_ and concentrated under reduced pressure. The oily product was finally washed with hexane to obtain the desired compound **2** as a brown oil (14.1 g, quantitative yield). ^1^H NMR (400 MHz, CDCl_3_) δ 7.40 (d, *J* = 1.5 Hz, 1H), 6.35 (d, *J* = 3.1 Hz, 1H), 6.33 (dd, *J* = 3.1, 1.5 Hz, 1H), 4.54 (s, 2H), 4.13 (d, *J* = 2.3 Hz, 2H), 2.46 (t, *J* = 2.3 Hz, 1H); ^13^C NMR (101 MHz, CDCl_3_) δ 150.9, 143.2, 110.5, 110.2, 79.4, 74.9, 63.2, 56.9. Spectroscopic data were in accordance with data from a previously reported synthesis [27].

**4,5-Dihydroisobenzofuran-5-ol (3). Adapted from reference** [19]**:** Compound **2** (14.1 g, 103.8 mmol) was added to a solution of *t*-BuOK (23.2 g, 207.5 mmol) in 100 mL of *t*-BuOH and heated at reflux for 5 hours. Then, 100 mL of water were added followed by multiple extractions with Et_2_O until a colorless organic phase was obtained. The combined organic phases were washed with brine, dried over MgSO_4_ and evaporated in vacuo*.* The product was then purified by flash chromatography using a SiO_2_ cartridge (80 g) with a gradient of hexane/AcOEt from 2:1 to 1:1 (TLC hexane/AcOEt 2:1 *R*_f_ = 0.4). Pure 4,5-dihydroisobenzofuran-5-ol (**3**) was obtained as a yellow oil (12.0 g, 85%). ^1^H NMR (400 MHz, CDCl_3_) δ 7.31 (s, 1H), 7.19 (s, 1H), 6.51 (d, *J* = 9.8 Hz, 1H), 5.92 (dd, *J* = 9.8, 3.9 Hz, 1H), 4.49 (td, *J* = 6.6, 3.9 Hz, 1H), 2.87 (dd, *J* = 15.7, 6.6 Hz, 1H), 2.77 (dd, *J* = 15.7, 6.6 Hz, 1H), 2.03 (s, 1H); ^13^C NMR (101 MHz, CDCl_3_) δ 138.3, 137.3, 129. 7, 120.4, 119.9, 117.3, 65.5, 27.9. Spectroscopic data were in accordance with data from a previously reported synthesis [28].

**5-Methoxy-4,5-dihydroisobenzofuran (4b):** Compound **3** (3.3 g, 24.3 mmol) was added to a solution of NaH (1.46 g, 36.4 mmol) in anhydrous dioxane (20 mL). After 15 minutes, methyl iodide (1.8 mL, 29.1 mmol) was slowly added and the resulting solution was stirred overnight at room temperature. The reaction was quenched by the addition of EtOH followed by water and the resulting heterogeneous mixture was filtered. The resulting solution was then dried over MgSO_4_ and concentrated under reduced pressure to obtain the title compound (3.4 g, 95%) as an orange oil used without further purification. IR (ATR, cm^−1^) 1075, 791, 586; ^1^H NMR (400 MHz, CDCl_3_) δ 7.30 (s, 1H), 7.18 (s, 1H), 6.55 (d, *J* = 9.9 Hz, 1H), 5.93 (dd, *J* = 9.9, 3.6 Hz, 1H), 4.12–4.11 (m, 1H), 3.39 (s, 3H), 2.90 (dd, *J* = 15.6, 6.1 Hz, 1H), 2.76 (dd, *J* = 15.6, 6.1 Hz, 1H); ^13^C NMR (101 MHz, CDCl_3_) δ 137.8, 137.1, 127.6, 120.7, 120.2, 117.8, 74.2, 56.0, 24.4.

**4-Methoxy-*ortho*-phthalaldehyde (5b):** A mixture of compound **4b** (3.4 g, 22.7 mmol) and DDQ (10.3 g, 45.3 mmol) in anhydrous dioxane (100 mL) was stirred at room temperature for 2 days. Excess of DDQ was eliminated by filtration and the remaining solid was washed with CH_2_Cl_2_. The liquid phase was dried over MgSO_4_ and concentrated under reduced pressure to obtain a residue which was purified by two successive flash chromatography runs. The first one was run on 100% DCM mobile phase in order to eliminate DDQ residue (*R*_f_ = 0.9) and the second with ethyl acetate/hexane (3:1) in order to separate 4-MeO-OPA (*R*_f_ = 0.6) from OPA (*R*_f_ = 0.9). The resulting oily brown product is finally recrystallized in hexane to afford **5b** as white needles (2.8 g, 63%). mp 41–42 °C (Lit. [29] mp 41–43 °C); ^1^H NMR (400 MHz, CDCl_3_) δ 10.67 (s, 1H), 10.34 (s, 1H), 7.94 (d, *J* = 8.5 Hz, 1H), 7.46 (d, *J* = 2.6 Hz, 1H), 7.22 (dd, *J* = 8.5, 2.6 Hz, 1H), 3.95 (s, 3H); ^13^C NMR (101 MHz, CDCl_3_) δ 192.0, 191.0, 163.9, 138.7, 134.6, 129.6, 118.9, 114.7, 56.0. Spectroscopic data were in accordance with data from a previously reported synthesis [30]

**4-Hydroxy-*ortho*-phthalaldehyde (6):** A mixture of compound **5b** (200 mg, 1.2 mmol), *p*-TsOH (1.68g, 9.8 mmol) and [Bmim]Br (4.0 g, 18.3 mmol) was placed in a sealed vial and irradiated under microwave irradiation for 30 minutes at 150 °C under strong agitation. After cooling, the mixture was vigorously extracted with Et_2_O overnight. The ether layer was separated and concentrated under vacuum to obtain crude compound **6** which was then crystallized in hexane to obtain yellow crystals (181 mg, quantitative yield). Mp: >85 ^o^C (dec.) (Lit. [17] mp: >85 °C); ^1^H NMR (400 MHz, CDCl_3_) δ 10.65 (s, 1H), 10.31 (s, 1H), 7.91 (d, *J* = 8.4 Hz, 1H), 7.43 (d, *J* = 2.6 Hz, 1H), 7.19 (dd, *J* = 8.4, 2.6 Hz, 1H); ^1^H NMR (400 MHz, DMSO) δ 11.00 (s, 1H), 10.49 (s, 1H), 10.27 (s, 1H), 7.92 (d, *J* = 8.4 Hz, 1H), 7.26 (d, *J* = 2.5 Hz, 1H), 7.17 (dd, *J* = 8.4, 2.5 Hz, 1H); ^13^C NMR (101 MHz, CDCl_3_) δ 192.2, 191.2, 160.7, 139.0, 135.4, 129.9, 120.2, 116.8; ^13^C NMR (101 MHz, DMSO) δ 193.8, 192.3, 162.8, 139.5, 134.9, 128.7, 120.4, 116.3. Data were in accordance with the literature [17].

## Supporting Information

File 1 Additional protocol and NMR characterization.
