# Supplementary material for: Efficient synthesis of 4-substituted-ortho-phthalaldehyde analogues: toward the emergence of new building blocks
Source: Beilstein J Org Chem. 2019 Mar 19;15:721–6. doi: 10.3762/bjoc.15.67 (PMC6444388; doi:10.3762/bjoc.15.67)
Supplement: File 1 — Additional protocol and NMR characterization. [file Beilstein_J_Org_Chem-15-721-s001.pdf]

BEILSTEIN JOURNAL OF ORGANIC CHEMISTRY

Supporting Information

for

Efficient synthesis of 4-substituted-*ortho*-phthalaldehyde analogues: toward the emergence of new building blocks

Clémence Moitessier, Ahmad Rifai, Pierre-Edouard Danjou, Isabelle Mallard and Francine Cazier-Dennin

Beilstein J. Org. Chem. **2019,** *15,* 721–726. doi:10.3762/bjoc.15.67

Additional protocol and NMR characterization

License and Terms: This is a supporting information file under the terms of the Creative Commons Attribution License (http://creativecommons.org/licenses/by/4.0). Please note that the reuse, redistribution and reproduction in particular requires that the authors and source are credited.
The license is subject to the *Beilstein Journal of Organic Chemistry* terms and conditions: (https://www.beilstein-journals.org/bjoc)

All the chemicals were purchased from Acros Organic or Sigma-Aldrich and were used as received without further purification.

Microwave assisted organic synthesis was carried using a 400 W Biotage initiator oven. Samples were placed in a sealed vial and irradiated with a temperature control.

Automated flash chromatography was performed on a Puriflash 430 (Interchim) incorporating a quaternary pump system, diode array and evaporation light scattering detectors using SiO_2 HC prepacked cartridge.

NMR spectra were recorded at 300 K with a Bruker Ascend 400 spectrometer operating at 400 MHz and 101 MHz for ^{1}H and ^{13}C, respectively. Traces of residual solvent were used as internal standard.

IR spectra were recorded using a Perkin–Elmer instrument equipped with an ATR module

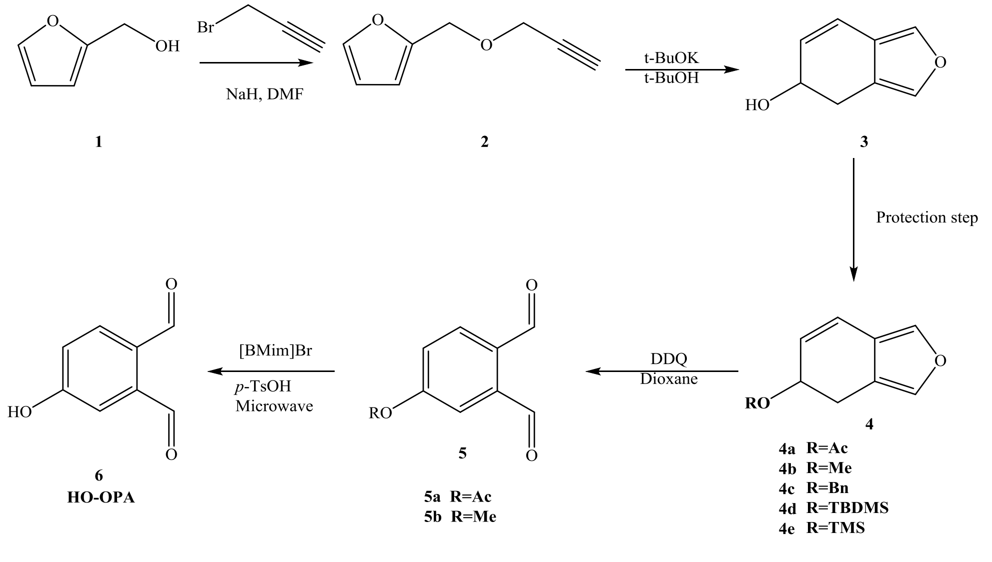

Scheme S1: General synthesis of 4-hydroxy-*ortho*-phthalaldehyde.

5-Acetoxy-4,5-dihydroisobenzofuran (4a)

4,5-Dihydrobenzofuran-5-ol (4.72 g, 34.7 mmol) was added to a mixture of acetic anhydride and pyridine (40:40; v:v; mL) and the resulting mixture was stirred into darkness during 72 hours at room temperature. Solvents were evaporated under reduced pressure. Remaining pyridine was eliminated by azeotropic distillation with toluene to afford the final product as a yellow oil (5.87 g, 95%). IR (ATR, cm^{-1}) 1709, 1234, 1001, 748, 602. ^{1}H NMR (400 MHz, $CDCl_3$) δ 7.33 (s, 1H), 7.18 (s, 1H), 6.60 (d, *J* = 9.8 Hz, 1H), 5.84 (dd, *J* = 9.8, 4.1 Hz, 1H), 5.52 (td, *J* = 6.2, 4.1 Hz, 1H), 2.87 (dd, *J* = 16.2, 6.2 Hz, 1H), 2.74 (dd, *J* = 16.2, 6.2 Hz, 1H), 1.93 (s, 3H). ^{13}C NMR (101 MHz, $CDCl_3$) δ 170.7 , 138.0 , 137.6 , 125.1 , 121.8 , 120.2 , 116.9 , 67.7 , 24.5 , 21.3.

5-(Benzyloxy)-4,5-dihydroisobenzofuran (4c)

A mixture of 4,5-dihydrobenzofuran-5-ol (0.15 g, 1.1 mmol) and NaH (0.09 g, 2.2 mmol) in 6 mL of anhydrous dioxane was stirred in darkness at room temperature during 30 minutes. Following the addition of benzyl chloride (0.26 mL, 2.2 mmol), the solution was left overnight under agitation. Then 10 mL of water was added and the aqueous phase was extracted with dichloromethane and ether. The combined organic layers were concentrated under reduce pressure to afford a brown oil (24 mg, 10%). ^{1}H NMR (400 MHz, $CDCl_3$) δ 7.42 – 7.34 (m, 5H), 7.32 (s, 1H), 7.20 (s, 1H), 6.53 (d, *J* = 9.6 Hz, 1H), 5.94 (dd, *J* = 9.6, 4.2 Hz, 1H), 4.59 (s, 2H), 4.50 (td, *J* = 6.2, 4.2, 1H), 2.96 (dd, *J* = 16.2, 6.2, 1H), 2.84 (dd, *J* = 16.2, 6.2, 1H).

***tert*-Butyl((4,5-dihydroisobenzofuran-5-yl)oxy)dimethylsilane (4d)**

Imidazole (1.6 g, 23.2 mmol) and *tert*-butyldimethylsilyl chloride (TBDMSCl) (2.1 g, 13.92 mmol) were added at 0 °C to a solution of 4,5-dihydrobenzofuran-5-ol (1.58 g, 11.6 mmol) in 50 mL of THF. The resulting mixture was stirred overnight at room temperature. The resulting solution was diluted with 25 mL of saturated ammonium chloride solution and extracted with Et_2O. The organic phases were combined and dried over $MgSO_4$. After solvent evaporation, yellow oil was obtained (0.88 g, 30%). ^{1}H NMR (400 MHz, $CDCl_3$) δ 7.24 (s, 1H), 7.12 (s, 1H), 6.37 (d, *J* = 9.8 Hz, 1H), 5.75 (dd, *J* = 9.8, 2.9 Hz, 1H),

4.60 (td, J = 6.3, 2.9 Hz, 1H), 2.64 (dd, J = 16.2, 6.3 Hz, 1H), 2.60 (dd, J = 16.2, 6.3 Hz, 1H), 0.93 (s, 9H), 0.12 (s, 6H).

((4,5-Dihydroisobenzofuran-5-yl)oxy)trimethylsilane (4e)

Imidazole (1.6 g, 23.2 mmol, 2 equiv) and trimethylsilyl chloride (TMSCl) (1.8 mL, 13.92 mmol, 1.2 equiv) were added at 0 °C to a solution of 4,5-dihydrobenzofuran-5-ol (1.58 g, 11.6 mmol) in 50 mL of THF. The resulting mixture was stirred overnight at room temperature. The obtain solution was diluted with 25 mL saturated ammonium chloride solution and extracted with Et_2O. Organic phases were combined and dried by $MgSO_4$. After solvent evaporation, yellow oil was obtained (0.37 g, 15%). ^{1}H NMR (400 MHz, $CDCl_3$) δ 7.32 (s, 1H), 7.13 (s, 1H), 6.39 (d, J = 9.7 Hz, 1H), 5.74 (dd, J = 9.7, 3.9 Hz, 1H), 4.57 (td, J = 6.4, 3.9 Hz, 1H), 2.87 (dd, J = 16.4, 6.4 Hz, 1H), 2.81 (dd, J = 16.4, 6.4 Hz, 1H), 0.11 (s, 9H).

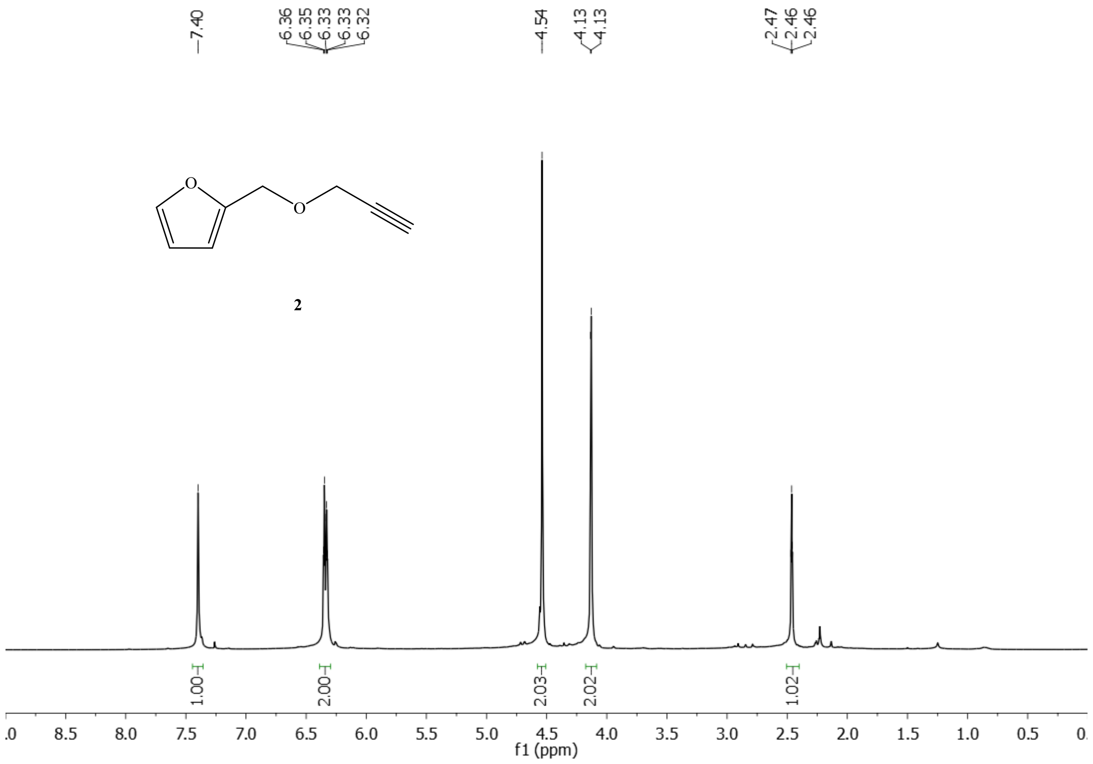

Figure S1: ^{1}H NMR spectrum (400 MHz, $CDCl_3$) of 2-((prop-2-ynyloxy)methyl) furan (compound 2).

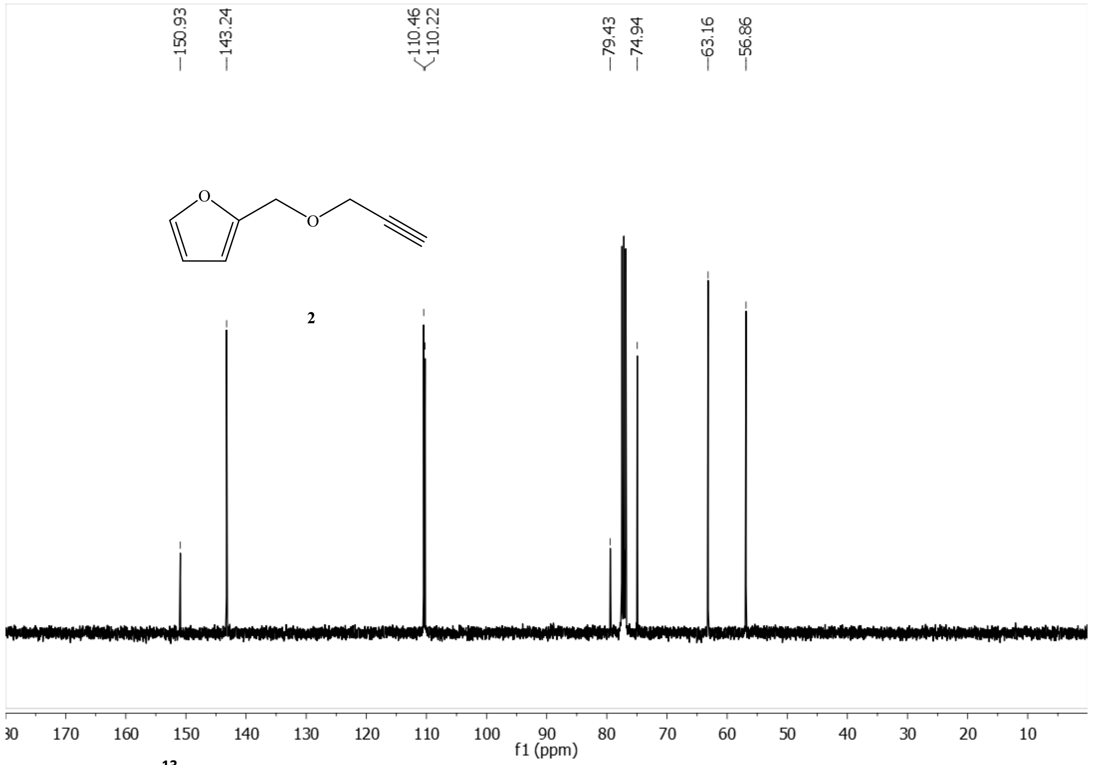

Figure S2: ^{13}C NMR spectrum (101 MHz, $CDCl_3$) of 2-((prop-2-ynyloxy)methyl) furan (compound 2).

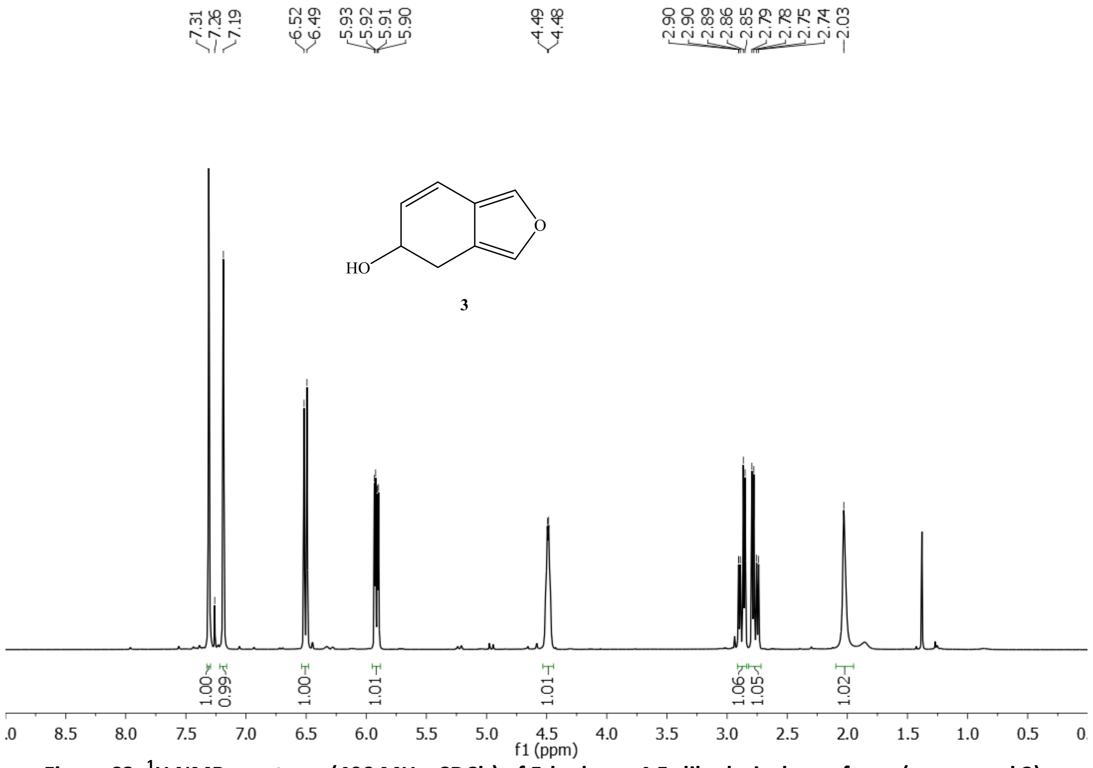

Figure S3: ^{1}H NMR spectrum (400 MHz, $CDCl_3$) of 5-hydroxy-4,5-dihydroisobenzofuran (compound 3).

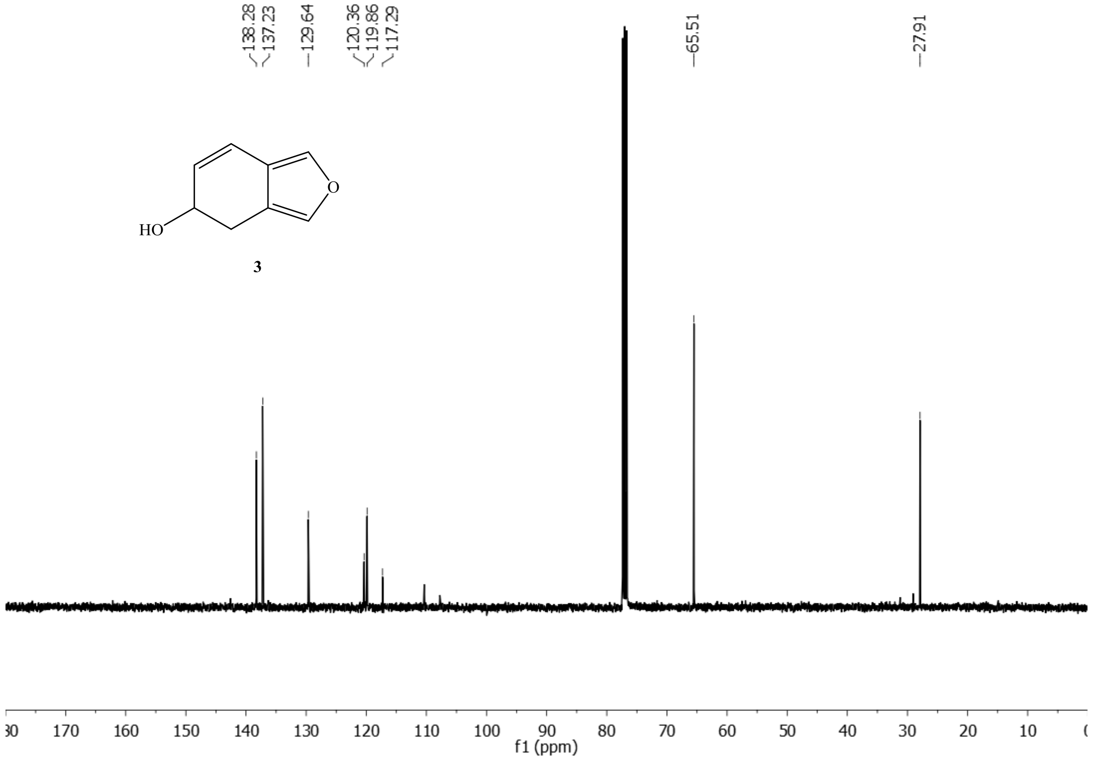

Figure S4: ^{13}C NMR spectrum (101 MHz, $CDCl_3$) of 5-hydroxy-4,5-dihydroisobenzofuran (compound 3).

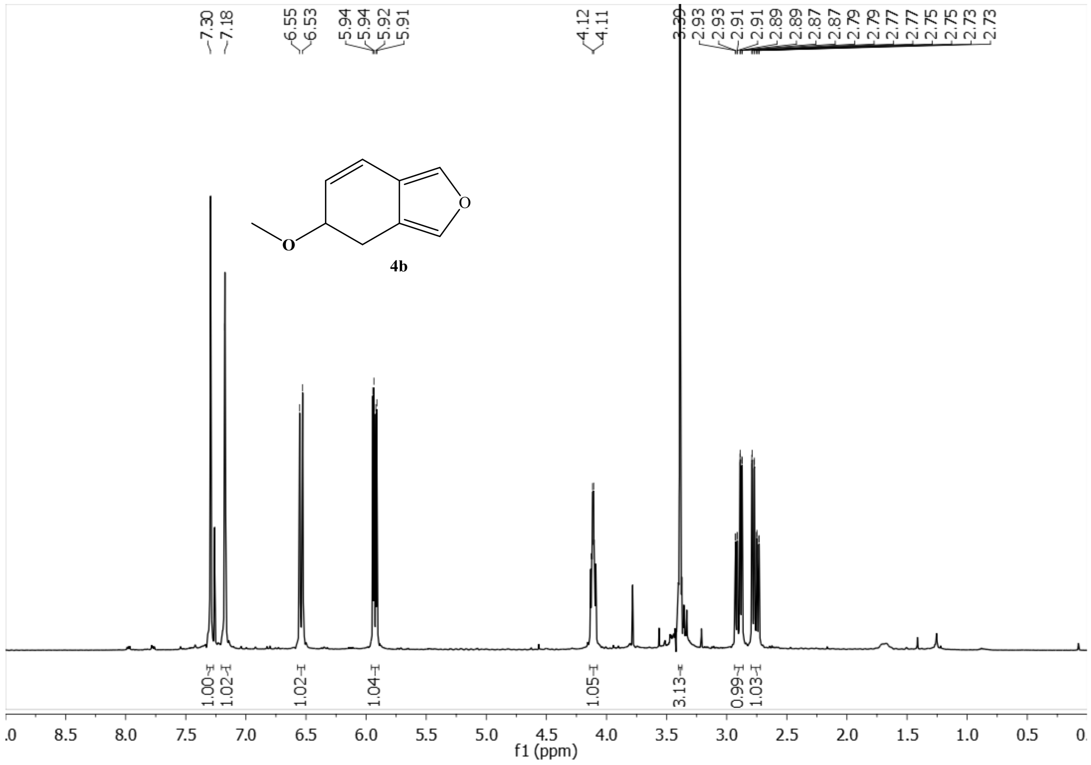

Figure S5: ^{1}H NMR spectrum (400 MHz, $CDCl_3$) of 5-methoxy-4,5-dihydroisobenzofuran (compound 4b).

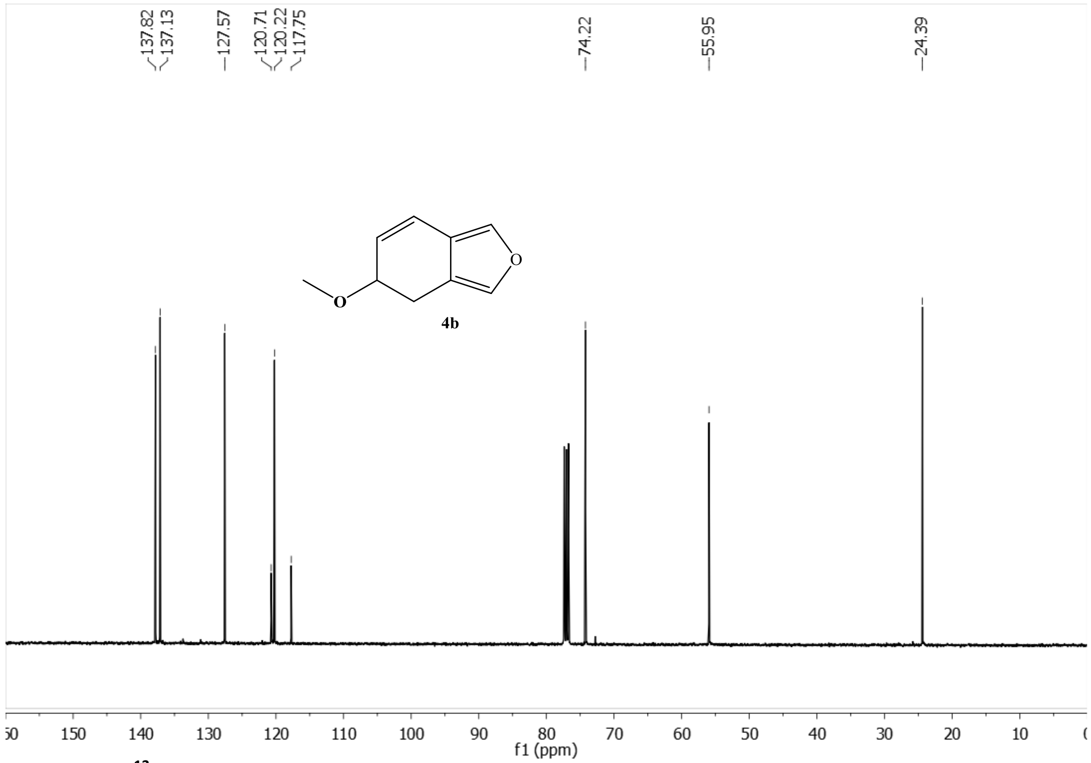

Figure S6: ^{13}C NMR spectrum (101 MHz, $CDCl_3$) of 5-methoxy-4,5-dihydroisobenzofuran (compound 4b).

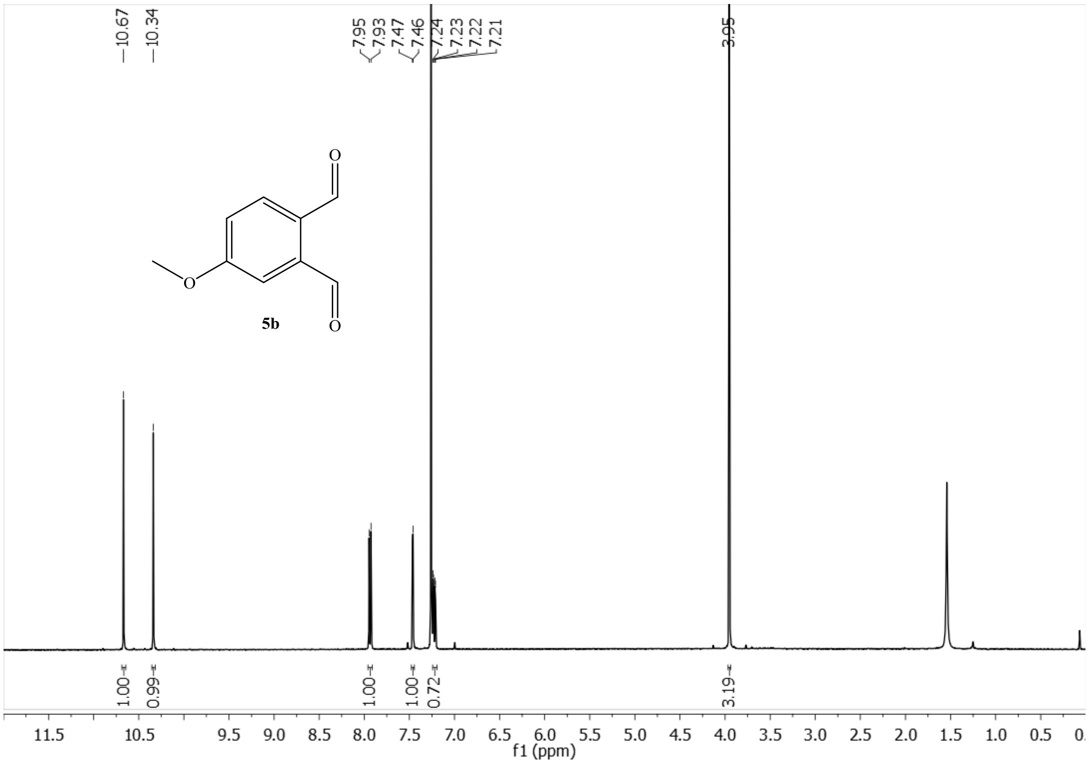

Figure S7: ^{1}H NMR spectrum (400 MHz, $CDCl_3$) of 4-methoxy-*ortho*-phthalaldehyde (compound 5b).

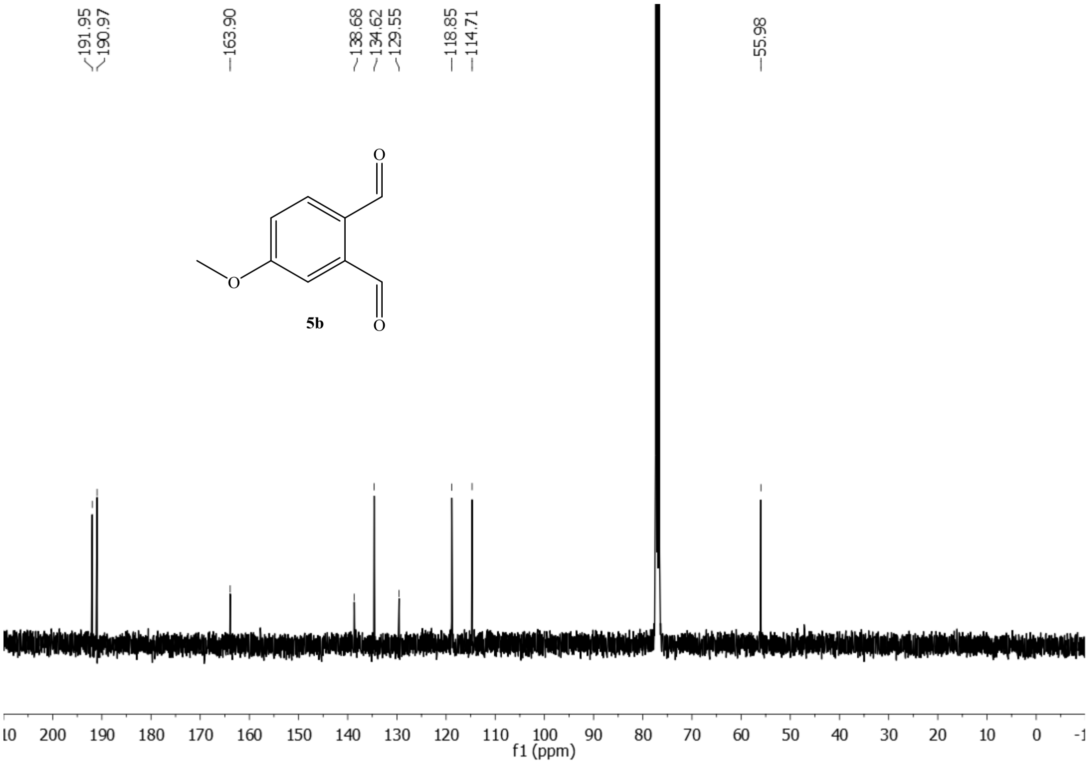

Figure S8: ^{13}C NMR spectrum (101 MHz, $CDCl_3$) of 4-methoxy-*ortho*-phthalaldehyde (compound 5b).

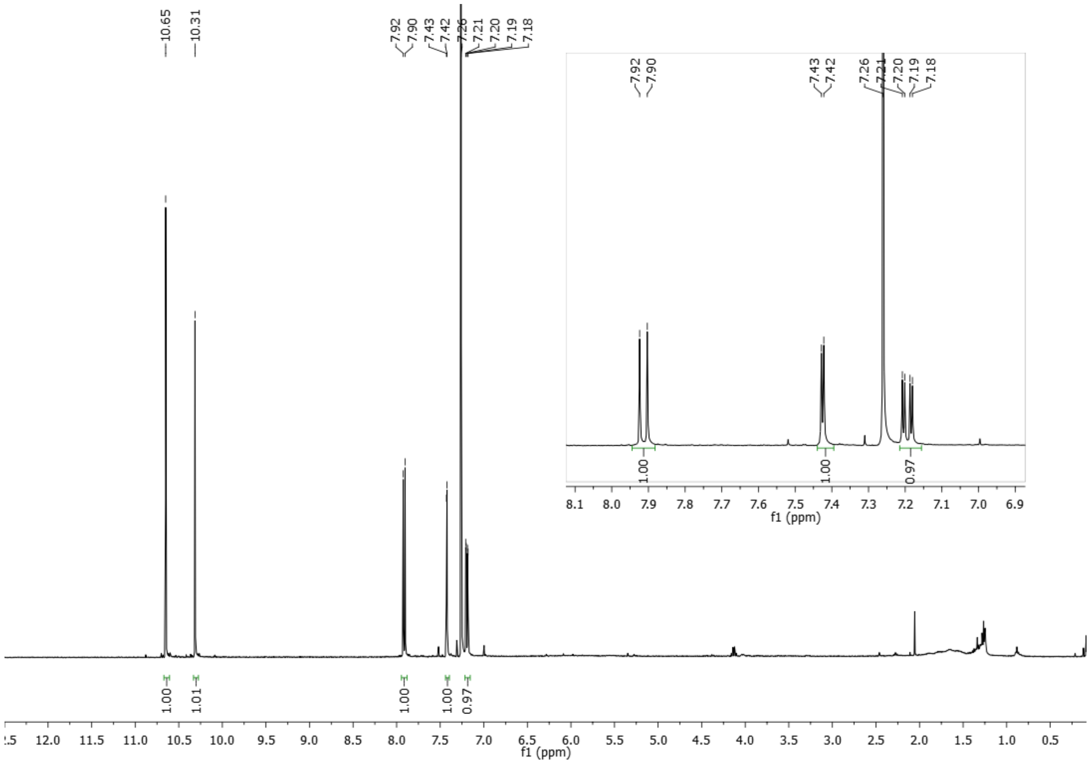

Figure S9: ^{1}H NMR spectrum (400 MHz, $CDCl_3$) of 4-hydroxy-*ortho*-phthalaldehyde (compound 6).

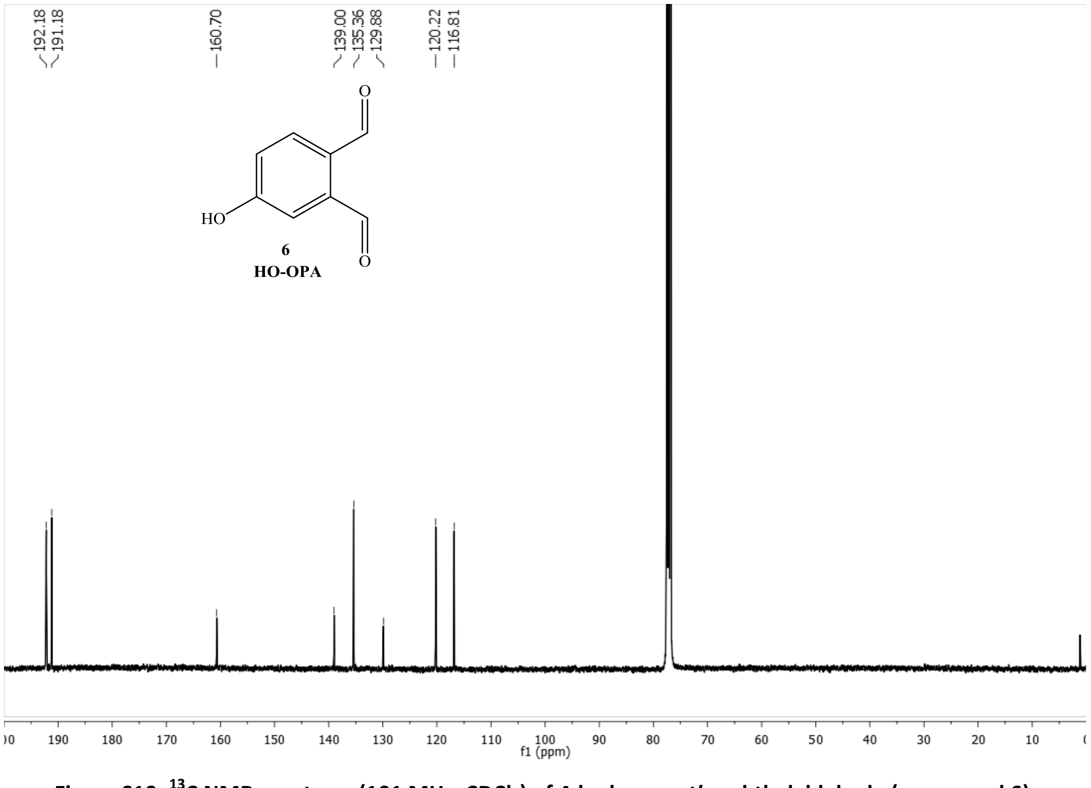

Figure S10: ^{13}C NMR spectrum (101 MHz, $CDCl_3$) of 4-hydroxy-*ortho*-phthalaldehyde (compound 6).
